# Exploring the Potential of Hop (*Humulus lupulus*) Cone Residue: Chemical Characterization and Evaluation of Bioactivities

**DOI:** 10.3390/plants15070994

**Published:** 2026-03-24

**Authors:** Giulia Boito Reyes, Emylaine Pereira dos Santos, Everton da Silva Santos, Laura Correia Gonçalves, Gabriela Catuzo Canonico Silva, Zilda Cristiani Gazim, Regina Aparecida Correia Gonçalves, Arildo José Braz de Oliveira, José Pinela, Filipa Mandim, Tânia C. S. P. Pires, Lucio Cardozo-Filho, Rúbia Carvalho Gomes Corrêa, José Eduardo Gonçalves

**Affiliations:** 1 Programa de Pós-Graduação em Tecnologias Limpas (PPGTL), e Instituto Cesumar de Ciência, Tecnologia e Inovação (ICETI), Universidade Cesumar (Unicesumar), Av. Guedner, 1610, Maringá 87050-390, Brazil; giuliaboito18@gmail.com (G.B.R.); emylainepereira4@gmail.com (E.P.d.S.); rubia.correa@unicesumar.edu.br (R.C.G.C.); 2 Programa de Pós-Graduação em Ciências Farmacêuticas (PCF), Universidade Estadual de Maringá (UEM), Av. Colombo, 5790, Maringá 87020-900, Brazil; everton.ds.santos@hotmail.com (E.d.S.S.); racgoncalves@uem.br (R.A.C.G.); ajboliveira@uem.br (A.J.B.d.O.); 3 Departamento de Medicina, Universidade Estadual do Centro-Oeste (Unicentro), Alameda Élio Antonio Dalla Vecchia, 838, Guarapuava 85040-167, Brazil; lauracorreiagoncalves@gmail.com; 4 Programa de Pós-Graduação em Biotecnologia Aplicada à Agricultura e Programa de Pós-Graduação em Ciência Animal com ênfase em Produtos Bioativos, Universidade Paranaense (Unipar), Praça Mascarenhas de Moraes, 4282, Umuarama 87502-210, Brazil; gabriela.canonico@edu.unipar.br (G.C.C.S.); cristianigazim@prof.unipar.br (Z.C.G.); 5 Centro de Investigação de Montanha, Associated Laboratory for Sustainability and Technology in Inland Regions (LA SusTEC), Instituto Politécnico de Bragança, Campus de Santa Apolónia, 5300-253 Bragança, Portugal; jose.pinela@iniav.pt (J.P.); filipamandim@ipb.pt (F.M.); tania.pires@ipb.pt (T.C.S.P.P.); 6 National Institute for Agricultural and Veterinary Research (INIAV, I.P.), Rua dos Lágidos, Lugar da Madalena, Vairão, 4485-655 Vila do Conde, Portugal; 7 Departamento de Engenharia Química, Universidade Estadual de Maringá (UEM), Av. Colombo, 5790, Maringá 87020-900, Brazil; lcfilho@uem.br

**Keywords:** hop cone residues, microwave- and ultrasound-assisted extraction, antioxidant bioactivity, pharmacological potential, green chemistry

## Abstract

*Humulus lupulus* L. (hops), belonging to the Cannabaceae family, is grown mainly for brewing, with 98% of global production directed to this sector. Moreover, large volumes of female cone residues are generated as by-products, representing a valuable source of bioactive compounds that can be valorized under green chemistry principles. This study aimed to extract bioactive compounds from hop cone residues sourced from craft breweries using ultrasound-assisted (EH-UA) and microwave-assisted (EH-MA) extraction methods. Hydroalcoholic extracts (70%) were analyzed for chemical composition, antioxidant, antimicrobial, antiproliferative, nitric oxide (NO)-production inhibition, and photoprotective activities. GC-MS identified 32 compounds in EH-MA and 30 in EH-UA, including terpenes, sesquiterpenes, oxygenated sesquiterpenes, and fatty acids. Both extracts demonstrated strong antioxidant activity in cell-based (TBARS, OxHLIA) and chemical (DPPH, ABTS, FRAP) assays, particularly EH-MA. Significant antibacterial activity was observed, especially against *Enterobacter cloacae*, *Pseudomonas aeruginosa*, and *Staphylococcus aureus* (MIC 1–10 mg/mL), as well as antifungal activity against *Aspergillus brasiliensis* (MIC 2–2.5 mg/mL). Selective antiproliferative activity was observed against tumor cell lines Caco-2 and MCF-7 (GI_50_ 25 μg/mL), without cytotoxicity toward nontumor cell lines Vero and PLP2 (GI_50_ > 400 μg/mL). All extracts inhibited the production of the inflammation mediator NO, with EH-MA showing the most potent effect (IC_50_ of 35 μg/mL), followed by EH-UA (IC_50_ of 55 μg/mL). Photoprotective potential was also demonstrated, with SPF values of 19 (EH-MA) and 18 (EH-UA). In conclusion, hop cone residues can yield multifunctional extracts with antioxidant, antimicrobial, antiproliferative, anti-inflammatory, and photoprotective activities, which support their sustainable upcycling for pharmacological, nutraceutical, and cosmetic applications.

## 1. Introduction

*Humulus lupulus L.*, commonly known as hops, is an angiosperm and a dioecious, herbaceous climbing plant of the Cannabaceae family that thrives in temperate regions worldwide, including Brazil [1,2]. Approximately 98% of global hop production is dedicated to the brewing industry, where secondary metabolites derived from female hop cones contribute to the bitterness, aroma, and preservation properties of beer [3,4]. Beyond brewing, hops have applications in pharmaceuticals, cosmetics, and nutraceuticals due to their diverse phytochemical properties [5]. The United States and Germany are the leading hop producers, with global production reaching 118,415 tons in 2023, an 11.5% increase from the previous year [6,7]. This large-scale production results in significant by-product residues that can be valorized through green chemistry principles.

Green chemistry promotes sustainable practices that minimize environmental impact. Brewery residues can be repurposed to produce organic fertilizers and animal feed. In addition, its bioactive compounds can be used in the food and pharmaceutical industries, reinforcing the role of circular economy practices in the beer production chain [8,9,10].

Conventional extraction methods (e.g., maceration, Soxhlet, or hydrodistillation) for bioactive compounds recovery are often inefficient and environmentally harmful, involving prolonged extraction times, large solvent volumes, potential thermal degradation of heat-sensitive compounds, and the use of toxic organic solvents or high-energy processes. In contrast, innovative techniques such as ultrasound- and microwave-assisted extraction offer quicker processes, reduced solvent use, lower energy consumption, and milder extraction conditions that preserve labile phytochemicals, thus offering a more sustainable and efficient alternative to conventional methods [11].

Therefore, this study investigates the effects of ultrasound- and microwave-assisted extraction on the chemical profile and biological activities of hop cone residues. The extracts’ chemical profile was investigated by GC-MS, whereas their biological potentials were assessed in terms of antimicrobial, antioxidant, antiproliferative, nitric oxide inhibition, and photoprotection properties.

## 2. Results and Discussion

### 2.1. Extraction Yield

The chemical composition and bioactive substances of cone residue from the Cascade variety of *Humulus lupulus* grown in Brazil were analyzed using environmentally friendly extraction methods: ultrasound-assisted extraction (UAE) and microwave-assisted extraction (MAE). The selection of the extraction solvent, specifically a 70% (*v*/*v*) hydroalcoholic solution, was based on physicochemical and environmental criteria to optimize process efficiency while minimizing environmental impacts. Ethanol and water are classified as sustainable solvents due to their low toxicity, high biodegradability, and lower environmental risk compared with conventional organic solvents [12].

The use of hydroalcoholic solutions promotes a synergistic effect between ethanol and water, allowing adjustment of the polarity of the extraction medium and consequently improving extraction efficiency. This synergy enhances both the diffusion of secondary metabolites from the plant matrix and the solubilization of compounds with different degrees of polarity [13].

Yields were determined by comparing the mass of the extract to the initial weight of the residue, resulting in 8.95% for EH-MA and 7.63% for EH-UA. The higher yield from EH-MA is attributed to rapid heating and effective cell wall disruption, while EH-UA enhances solvent diffusion through acoustic cavitation. Both methods improve extraction efficiency over conventional techniques by reducing solvent use and processing time while preserving heat-sensitive compounds [10].

### 2.2. Chemical Characterization by GC-MS

Table 1 compiles the main compounds identified in *H. lupulus* cone residue extracts by gas chromatography, along with their respective relative areas. A total of 55 compounds were identified in the extracts. GC-MS (Figure S1) detected 32 compounds for the EH-MA extract, whereas 30 components were detected for the EH-UA extract, including monoterpenes, sesquiterpenes, oxygenated sesquiterpenes, and fatty acids.

The major compounds identified in the EH-UA extract were β-pinene (**1**), caryophyllene (**35**), *cis*-β-farnesene (**37**), γ-muurolene (**40**), and azulene(1,2,3,3a,4,5,6,7-octahydro-1,4-dimethyl-7-(1-methyleneyl)-,[1R-(1α.3aβ.4α.7β)]) (**41**). For the EH-MA extract, β-pinene (**1**), caryophyllene (**35**), β-cubebene (**36**), geranyl propionate (**39**), and cadine-1(10),4-diene (**42**) were the leading phytochemicals. Some compounds were identified only in specific extracts, demonstrating that the methods present important conditions for the extraction of the compounds.

The chemical profile obtained in this study mainly represents the volatile fraction of the extract of hop (*H. lupulus*) cone residue identified by GC-MS [14,15,16]. However, we want to highlight the use of a byproduct that the brewing industry would otherwise discard. This byproduct can be used for other purposes, as it still contains many bioactive compounds.

Although the biological assays were conducted using the crude extract, the presence of major constituents such as β-pinene and cis-β-farnesene may contribute to the observed bioactivities. β-Pinene has been widely reported to exhibit anti-inflammatory, antimicrobial, and myorelaxant properties, including the inhibition of inflammatory mediators and leukocyte migration [17,18]. In addition, β-farnesene has shown promising biological potential, including affinity for inflammatory targets such as cyclooxygenase-2 (COX-2) and tumor necrosis factor-α (TNF-α) in molecular docking studies [19], as well as antiproliferative activity against Caco-2 colorectal adenocarcinoma cells and neuroprotective effects in SH-SY5Y neuroblastoma cells [20]. These findings suggest that the biological activities observed in the hop cone residue extract may be partly associated with these terpenoid compounds, although synergistic interactions among the extract constituents may also play an important role.

### 2.3. Antioxidant Activity

The antioxidant potential of hop cone residue extracts obtained via ultrasound-assisted (EH-UA) and microwave-assisted (EH-MA) methods was evaluated using both chemical assays (DPPH, FRAP, and ABTS) and cell-based in vitro assays (OxHLIA and TBARS). These methodologies are commonly used to estimate the antioxidant activity of plant-derived extracts, and the results are summarized in Table 2.

Among the chemical assays, the DPPH and ABTS methods exhibited the highest sensitivity to the extracts, yielding the most significant results for antioxidant potential (Table 2). The ultrasound-assisted extract (EH-UA) demonstrated the highest antioxidant potential, with 1425, 1234, and 944 µmol TE/mg DW (Dry Weight) for the DPPH, ABTS, and FRAP assays. The microwave-assisted extract (EH-MA) followed closely, with 1139 µmol TE/mg DW values for DPPH, 1097 µmol TE/mg DW for ABTS, and 928 µmol TE/mg DW for FRAP. These findings indicate that both extraction methods effectively recovered antioxidant compounds, with EH-UA showing a slight edge in overall performance.

The antioxidant potential of the extracts was further validated in cell-based assays. In the TBARS assay, EH-UA exhibited the strongest activity with an IC_50_ of 2.44 mg/mL, followed by EH-MA with an IC_50_ of 3.67 mg/mL. Despite this activity, none of the extracts exhibited an IC_50_ value lower than the positive control Trolox (IC_50_ of 0.0058 mg/mL). The TBARS assay, which simulates free radical-induced damage to cellular membranes, is a critical parameter for evaluating the ability of natural products to mitigate oxidative stress [21].

In the OxHLIA assay, EH-MA displayed an IC_50_ value of 22.50 µg/mL, followed by EH-UA with 31.30 µg/mL. Although these values were slightly less effective than the Trolox control (IC_50_ of 21.80 µg/mL), they highlight the extract’s protective effects against free radical-mediated erythrocyte membrane damage [22].

Our results are consistent with previous studies that reported antioxidant activity in *H. lupulus* extracts using DPPH, FRAP, ABTS [23,24,25], TBARS, and OxHLIA assays [14]. The antioxidant potentials herein verified suggest that extracts from hop cone residue exhibit a diverse chemical composition and can neutralize free radicals. This chemical diversity underscores the potential of these extracts for applications in mitigating oxidative stress-related conditions. Furthermore, it is worth nothing that other classes of bioactive compounds, not identified in this study (such as polyphenols) are likely implied with our extracts’ antioxidant potentials.

Similar antioxidant properties have been reported in the literature for different hop cultivars [26,27,28]. Extracts from the Cascade variety exhibited strong radical-scavenging activity, with ABTS values of 5.11 mM Trolox equivalents per mg of crude extract and DPPH activity of 1.34 mg mL^−1^, while FRAP activity reached 2.52 µM ferrous sulfate per mg for the Comet variety [26]. Significant free radical-scavenging activity has also been reported with IC_50_ values of 574.1 µg mL^−1^ for DPPH and 311.1 µg mL^−1^ for ABTS, demonstrating a concentration-dependent inhibitory effect [28]. In addition, variability in antioxidant activity among hop cultivars has been observed, with DPPH radical inhibition ranging from 0.31 to 0.98 mmol Trolox g^−1^ dry weight for Lubelski extracts, while ABTS scavenging activity ranged from 1.32 mmol Trolox g^−1^ dry weight for the Magnum cultivar to 2.43 mmol Trolox g^−1^ dry weight for the Marynka cultivar [27].

### 2.4. Antimicrobial Activity

The extracts demonstrated moderate inhibitory activity against both Gram-positive and Gram-negative bacteria, with greater effectiveness against Gram-negative strains, consistent with findings by [14] regarding *H. lupulus* cone residue. Such differential susceptibility between Gram-positive and Gram-negative bacteria is attributed to the structural differences in their outer membranes. Specifically, the unique outer membrane of Gram-negative bacteria serves as an effective barrier, limiting the penetration of many antimicrobial agents [29]. In addition to antibacterial effects, the extracts also exhibited antifungal activity, as summarized in Table 3.

The microwave-assisted extract (EH-MA) showed superior antimicrobial efficiency compared to the ultrasound-assisted extract (EH-UA), with greater inhibitory effects towards *Enterobacter cloacae* (MIC = 1 mg/mL), *Pseudomonas aeruginosa, Salmonella enterica* and *Staphylococcus aureus* (MIC = 2.5 mg/mL), besides *Bacillus cereus* (MIC = 5 mg/mL). However, the ultrasound-assisted extract (EH-UA) showed significant inhibitory activities against *E. cloacae*, *P. aeruginosa*, and *S. aureus* (MIC= 5 mg/mL), and against *Y. enterocolitica* and *E. coli* (MIC = 10 mg/mL).

Both extracts had comparable minimum bactericidal concentrations (MBCs) for *E. cloacae* and *S. aureus* (5–10 mg/mL). They showed antifungal activity against *Aspergillus brasiliensis*, with MICs of 2.0 mg/mL for EH-MA and 2.5 mg/mL for EH-UA (Table 3).

These results emphasize the antimicrobial potential of *H. lupulus* cone residue extracts. Although hop cone residues remain underexplored as sources of antimicrobial agents, this study provides valuable insights into their bioactive properties. The data obtained underscores the importance of further research to fully realize the potential applications of these extracts in combating microbial infections.

### 2.5. Antiproliferative Activity

To evaluate the safety of hop cone residue (*H. lupulus*) extracts, primary culture of pig liver cells (PLP2) and green monkey kidney cells (Vero) were tested. Results (Table 4) indicated that extracts EH-UA and EH-MA did not exhibit the capacity to inhibit non-tumor cells’ proliferation at the tested concentrations (GI_50_ > 400 μg/mL). These findings suggest a favorable safety profile for the extracts in non-tumor cells.

The extracts effectively inhibited tumor cell proliferation in AGS, Caco-2, and MCF-7 cells, with GI_50_ values ranging from 25 to 100 µg/mL. Notably, EH-MA was particularly effective against Caco-2 cells (GI_50_ of 25 µg/mL), while EH-UA showed better activity against MCF-7 cells (GI_50_ of 25 µg/mL). When the cytotoxicity of the extracts was evaluated in VERO cells, both presented GI_50_ values > 400 µg/mL. Reference criteria used to classify cytotoxicity in VERO cells indicate that a substance is considered cytotoxic when CC_50_ < 100 µg/mL, moderately cytotoxic when CC_50_ ranges between 100 and 200 µg/mL, and non-cytotoxic when CC_50_ > 200 µg/mL [30]. Accordingly, the EH-UA and EH-MA extracts did not exhibit cytotoxicity.

The selectivity index (SI), which measures the toxicity of samples toward tumor cells over non-tumor cells, indicated promising results for both extracts. EH-MA showed the highest SI of 16 for Caco-2, 8 for AGS, and 4 for MCF-7 cells, while EH-UA had an SI of 16 for MCF-7, 8 for AGS, and 4 for Caco-2 cells (Table 4). These results underscore the selective antitumor potential of the extracts.

Previous studies have reported antitumor activity in extracts of hop cones [25,31]. However, this study is the first to report the antiproliferative activity of hop cone residue extracts. This finding contrasts with previous reports indicating that supercritical fluid extracts of hop cone residue did not exhibit antitumor activity [14].

These findings suggest that hop cone residue extracts can be safely integrated into products for human consumption. However, further rigorous testing is essential to confirm their safety and efficacy for pharmaceutical and nutraceutical applications. This analysis is critical for assessing the therapeutic potential of these extracts and understanding their safety profile in healthy cells.

To evaluate the effectiveness of the tested extracts, we compared their results with those of ellipticine, a well-known cytotoxic agent widely referenced in the scientific literature. The results indicate that hop cone residue extracts exhibit significant antitumor activity and a favorable safety profile in non-tumor cells.

### 2.6. NO-Production Inhibition

The capacity of the studied extracts to inhibit nitric oxide production using a lipopolysaccharide (LPS)-stimulated murine macrophage cell was tested; the results obtained are shown in Table 5.

All extracts from hop cone residue (*H. lupulus*) inhibited NO production. The most significant effect was observed with extract EH-MA, which exhibited the lowest IC_50_ value (35 µg/mL), followed by EH-UA (55 µg/mL). Nitric oxide is a key mediator of the inflammatory process, mainly produced by inducible nitric oxide synthase (iNOS) in stimulated macrophages. Therefore, the inhibition of NO production indicates that the studied extracts may interfere with inflammatory signaling pathways associated with iNOS expression. 

These results align with findings described in previous studies, reporting the anti-inflammatory potential of *H. lupulus* [16,32,33]. However, to the best of our knowledge, this is the first report documenting the activity of the byproduct derived from the cone residue of *H. lupulus*. Further studies should focus on elucidating the molecular mechanisms underlying the observed activity, as and on identifying the bioactive compounds responsible for these effects [34,35].

### 2.7. Photoprotective Activity

The photoprotective activity of hop cone residue extracts, specifically EH-UA and EH-MA, was evaluated by determining their Sun Protection Factor (SPF) across various concentrations, as shown in Table 6. Both extracts demonstrated significant potential for UV protection, with EH-MA exhibiting SPF values of 18.40–18.80 (approximately 19 times greater protection than untreated skin) and EH-UA showing SPF values of 17.50–17.90 (about 18 times more protection). These findings highlight the robust UV-protective properties of both extracts, with EH-MA displaying slightly greater efficacy.

These results underscore the extracts’ efficacy as natural sunscreens, aligning with existing literature on plant-derived compounds [36]. Although consistent SPF values were observed at different concentrations, the extrapolation of these findings to cosmetic or dermatological applications should be interpreted with caution, as such an inference may still be considered premature. Therefore, the data obtained should be regarded as preliminary evidence of the photoprotective potential associated with compounds derived from *H. lupulus*. In this context, further studies are required to confirm and more comprehensively characterize this potential, including the evaluation of the UVA/UVB ratio, photostability analyses, and investigations using cellular models of UV radiation–induced damage. Nevertheless, the results presented here contribute to expanding current knowledge on plant-derived photoprotective agents and indicate promising perspectives for future studies exploring the valorization of *H. lupulus* by-products in the development of skin-care formulations.

## 3. Materials and Methods

### 3.1. Plant Materials

The Cascade variety of hops (*H. lupulus* L.) used in this study was sourced from Brazilian cultivation in Lages, Santa Catarina, Brazil, located at the coordinates 27°76′89.1″ S 50°27′63.8″ W. A craft brewery in Maringá, Paraná, Brazil, supplied the hop cone residue. This residue underwent lyophilization (JJ Científica model LJJ, São Carlos, Brazil), was crushed to a particle size of approximately 1.20 mm (Tyler mesh 16) and was stored at −26 °C.

### 3.2. Extraction Procedures

#### 3.2.1. Ultrasound-Assisted Extraction

The ultrasound-assisted extraction (UAE) was adapted from [10], where 2 g of hops cone residue was mixed with 20 mL of a 70% hydroalcoholic solvent (1:10, *w*/*v*). The extraction process was conducted using an ultrasonic extractor (Optic Ivymen System CY-500, Barcelona, Spain) set at 30 kHz and a temperature of 35 °C for 35 min. After processing, the resulting extract (denoted as EH-UA, for hop residue extract obtained by ultrasound-assisted extraction) was centrifuged for 30 min at 4000 rpm (Kasvi, model 4000 centrifuge, Pinhas, Brazil). The supernatant was then separated and concentrated under reduced pressure using a rotary evaporator at 30 °C (Fisaton, São Paulo, Brazilm, model Fisaton 802). Finally, the extract was lyophilized at −86 °C (JJ Científica, model LJJ, São Carlos, Brazil) and stored for analysis.

#### 3.2.2. Microwave-Assisted Extraction

The microwave-assisted extraction (MAE) was adapted from [11], where 2 g of hop cone residue samples were suspended in 20 mL of a 70% hydroalcoholic extractant solution and processed in a microwave oven with a rotating base (Provecto Analítica, model DGT 100-Plus, Jundiai, Brazil). The microwave program consisted of an initial 1 min of extraction at 200 W, followed by 5 min at 400 W, then 1 min at 600 W, and finishing with 5 min at 0 W. After extraction, the resulting extract (EH-MA: extract of hop residue by microwave) was centrifuged for 30 min at 4000 rpm (Kasvi model 4000 centrifuge). The supernatant was separated, concentrated under reduced pressure (Fisaton 802), lyophilized at −86 °C (JJ Científica, model LJJ, São Carlos, Brazil) and stored until analysis.

### 3.3. Chemical Characterization by GC-MS

The extracts were analyzed using a GC-MS (Agilent, model 7890B—Agilent, model 5977A MSD, Santa Clara, CA, USA), employing an HP-5MS IU capillary column (Agilent). A 2 μL aliquot of the sample diluted (100 mg of the extract in 2 mL of ethyl acetate containing 2.5 μg/mL internal standard) was injected. The oven was set to an initial temperature of 50 °C and maintained for 1 min. It was then heated at 10 °C/min to 250 °C, It was then heated at a rate of 10 °C/min to 250 °C, finishing with heating at 25 °C/min to 300 °C where it remained for 1 min. Sample injection was performed in split mode at a 1:20 dilution ratio, with a constant helium flow of 1.0 mL/min. The injector and transfer line temperatures were 250 °C and 290 °C, respectively. In the mass detector, the ionization source and quadrupole temperatures were 230 °C and 150 °C. The mass spectrometer utilized an electron multiplier (EM) detector in “scan” mode, with a mass-to-charge ratio (*m*/*z*) range set from 40 to 600. To ensure accurate measurements, a solvent delay of 3 min was implemented.

Compounds were identified by comparing their mass spectra with those from the NIST 11.0 library and their retention indices (RI), which were determined using a homologous series of n-alkane standards ranging from C7 to C40. The concentrations of the compounds were calculated by comparing the relative areas of each chromatographic peak to the peak area of the octadecane internal standard (Sigma-Aldrich, St. Louis, MO, USA) at a concentration of 2.5 μg/mL in ethyl acetate.

### 3.4. Bioactive Properties

#### 3.4.1. Antioxidant Capacity

*Ferric reducing antioxidant power (FRAP):* A volume of 100 μL of the samples in concentration of 1 mg/mL (diluted in methyl alcohol) were mixed with 300 μL deionized H_2_O and 3 mL of the FRAP reagent (0.3 M acetate buffer, 10 mM TPTZ (2,4,6-*Tris* (2-pyridyl)-s-triazine) solution, 20 mM ferric chloride solution; 10:1:1, *v*/*v*/*v*) for 30 min at 37 °C. Then, the absorbances was measured on the spectrophotometer (Agilent Cary 60 UV-Vis, Santa Clara, CA, USA) at 593 nm, against a blank consisting of methyl alcohol and sample concentrations were calculated using a standard curve obtained for standard solutions prepared in the same way as the analyzed samples, but in which Trolox was used as the standard [14]. The results were expressed in µmol Trolox equivalent (TE)/mg extract dry weight (DW).

*DPPH radical-scavenging activity*: Experimental data on antioxidant activity were obtained using the 2,2-diphenyl-1-picrylhydrazyl (DPPH) test. The samples (volume of 25 μL; in concentration of 1 mg/mL, diluted in methyl alcohol) were mixed with 2 mL of the 6.25 × 10^−5^ mol/L DPPH^•^ solution for 30 min, Then, the absorbance was measured on the spectrophotometer (Agilent Cary 60 UV-Vis, Santa Clara, CA, USA) at 517 nm against a blank consisting of methyl alcohol and sample concentrations were calculated using a standard curve obtained for standard solutions prepared in the same way as the analyzed samples, but in which Trolox was used as the standard [37]. The results were expressed in µmol Trolox equivalent (ET)/mg extract dry weight (DW) and the ascorbic acid (Sigma-Aldrich) was used as a positive control.

*ABTS radical-scavenging activity*: This assay was carried out using the 2,2′-azino-*bis*(3-ethylbenzothiazoline-6-sulfonic acid), ABTS. The samples (volume of 30 μL) in concentration of 1 mg/mL diluted in methyl alcohol) were mixed with 3 mL of the ABTS^•+^ solution (5 mL of a 7 mmol/L ABTS solution and 88 μL of a 140 mmol/L potassium persulfate; reaction for 16 h) for 6 min, and the absorbance was measured on the spectrophotometer (Agilent Cary 60 UV-Vis, Santa Clara, CA, USA) at 734 nm against a blank consisting of methyl alcohol and sample concentrations were calculated using a standard curve obtained for standard solutions prepared in the same way as the analyzed samples, but in which Trolox was used as the standard [14]. The results were expressed in µmol Trolox equivalent (TE)/mg extract dry weight (DW) and the ascorbic acid (Sigma-Aldrich, St. Louis, MO, USA) was used as a positive control.

*Oxidative hemolysis inhibition assay (OxHLIA):* An erythrocyte solution (2.8%, *v*/*v*; 200 µL) prepared in phosphate-buffered saline (PBS, pH 7.4) was mixed with 400 µL of either: (i) tested extracts at different concentrations (4–512 µg/mL in PBS); (ii) distilled water (baseline); (iii) PBS (negative control); or (iv) Trolox (7.81–250 µg/mL in PBS). After preincubation at 37 °C for 10 min with shaking, 200 μL of 2,2′-azobis(2-methylpropionamidine) dihydrochloride (AAPH, 160 mM; Sigma-Aldrich, St. Louis, MO, USA) was added, and the optical density was measured at 690 nm (Bio-Tek Instruments, ELX800, Winooski, VT, USA) every 10 min until complete hemolysis [38]. IC_50_ values (µg/mL) were calculated for a 60 min Δt, corresponding to the sample concentration required to protect 50% of the erythrocyte population from the hemolytic action of AAPH for a 60 min time interval, and the Trolox (Sigma-Aldrich, St. Louis, MO, USA) was used as a positive control [37].

*Thiobarbituric acid reactive substances formation inhibition (TBARS):* To evaluate the extract’s capacity to inhibit lipid peroxidation, 200 µL of different concentrations of the extracts dissolved in water (between 5 and 0.156 mg/mL) were incubated for 1 h at 37 °C with ascorbic acid (100 µL; 0.1 mM)), iron sulfate (100 µL; 10 µM), and brain tissue suspension (100 µL; prepared in Tris-HCl, 20 mM, pH 7.4, at a ratio of 1:2 *w*/*v*). Subsequently, 500 μL of trichloroacetic acid (28% *w*/*v*) and 380 μL of thiobarbituric (2% *w*/*v*) were added. After incubation at 80 °C for 20 min, the malondialdehyde–thiobarbituric acid complex was determined by measuring the absorbance at 532 nm, against a blank where the extract was replaced by the corresponding solvent (i.e., water and Tris-HCl solution). The results were expressed as IC_50_ values (μg/mL), and the Trolox (Sigma-Aldrich, St. Louis, MO, USA) was used as a positive control [14]. The IC_50_ value was determined from the dose–response curve obtained by plotting the percentage of inhibition against the sample concentration.

#### 3.4.2. Antimicrobial Capacity

The extracts of *H. lupulus* cone extracts were dissolved in 5% (*v*/*v*) dimethyl sulfoxide (DMSO) to obtain a stock solution at 20 mg/mL, which was serially diluted to obtain different concentrations (10–0.15 mg/mL). The antibacterial activity was tested against five Gram-negative bacteria strains (*Enterobacter cloacae* ATCC 49741, *Escherichia coli* ATCC 25922, *Pseudomonas aeruginosa* ATCC 9027, *Salmonella enterica* subsp. *enterica* ATCC 13076, and *Yersinia enterocolitica* ATCC 29906) and three Gram-positive strains (*Bacillus cereus* ATCC 11778, *Listeria monocytogenes* ATCC 19111, and methicillin-resistant *Staphylococcus aureus* MRSA, ATCC 25923). The antifungal activity was tested against two fungal species (*Aspergillus fumigatus* ATCC 204305, and *Aspergillus brasiliensis* ATCC 16404).

The minimum inhibitory concentrations (MICs) were determined using the serial microdilution method and the *p*-iodonitrotetrazolium chloride (INT) rapid colorimetric assay [39]. MICs values were defined as the lowest extract concentration that inhibits visible bacterial growth. For fungi, it was the lowest concentration at which no visible growth was observed. The MBC/MFC values correspond to the lowest concentration at which no microorganism growth occurred.

Two negative controls were tested, one with tryptic soy broth (TSB) and another with extract. Two positive controls were prepared with TSB and each inoculum and another with medium, antibiotics (Streptomycin, ampicillin, and methicillin) or fungicide (ketoconazole), and bacteria/fungi. Results were presented as MICs and MBC or MFCs values in mg/mL.

#### 3.4.3. Antiproliferative Activity

The antiproliferative activity of the studied extracts was tested in several tumor and non-tumor cell lines. The human tumor cell lines of gastric (AGS), colorectal (Caco-2), and breast adenocarcinomas (MCF-7) were tested, as well as the non-tumor African green monkey kidney (Vero) and the primary culture from pig liver (PLP2), All cell lines were maintained in Roswell Park Memorial Institute (RPMI) 1640 medium supplemented with 10% heat-inactivated fetal bovine serum (FBS), non-essential amino acids (2 mM), L-glutamine (2 mM), penicillin (100 U/mL), and streptomycin (100 μg/mL), except Vero cells that were maintained with Dulbecco’s Modified Eagle Medium (DMEM) supplemented as mentioned above.

The extracts were dissolved in water and successively diluted to obtain different concentrations (125–8000 μg/mL). Each concentration (10 μL) was incubated with 190 μL of the cell suspension (10,000 cells/well, except for the Vero cells, which were tested at 19,000 cells/well) in a 96-well microplate for 72 h. The cellular protein content was determined by the sulforhodamine B (SRB) assay. The absorbance was read at 540 nm using the BioTek ELx800 microplate reader mentioned above [40]. Ellipticine was tested as the positive control, while the cells without extract were the negative control. All experiments were performed in triplicate in three independent assays. The results were expressed as the sample concentration required to inhibit cell proliferation by 50% (GI_50_, μg/mL).

#### 3.4.4. Nitric Oxide (NO)-Production Inhibition

The NO-production inhibition capacity was assessed in a lipopolysaccharide (LPS)-stimulated murine macrophage cell line (RAW 264.7) [40]. The extracts were redissolved in water (8 mg/mL) and successively diluted to obtain the range of concentrations to be tested (final concentrations tested between 400 and 6.25 μg/mL). The NO concentration was determined using the Griess Reagent System kit. The nitric oxide produced was quantified by measuring the absorbance at 540 nm with the BioTek microplate reader mentioned above and comparing the results with the standard calibration curve (nitrite curve prepared using sodium nitrite concentrations from 100 mM to 1.6 mM). Dexamethasone was utilized as a positive control, and cells with and without LPS were tested as negative controls. All experiments were performed in triplicate in three independent assays. The results are expressed as IC_50_ values (μg/mL), representing the sample concentration needed to achieve 50% inhibition of NO production.

#### 3.4.5. Photoprotective Capacity

The photoprotective activity was evaluated in vitro by determining the maximum wavelength (λmax) and maximum absorbance (Amax). The extracts were diluted in ethyl alcohol PA (Merck) (50–1000 μg/mL, *w*/*v*) and scanning was performed between wavelengths of 260 to 400 nm (Agilent Spectrophotometer, Cary 60, Santa Clara, CA, USA) to verify absorption in the ultraviolet A and B regions (UVA and UVB).

The sun protection factor (SPF) was calculated based on the method described by Orlanda and Vale [41]. The erythemogenic effect of radiation (EE(λ)) and solar intensity (I(λ)) values were obtained from standard reference data, applying the following equation:

$$Sun\;Protection\;Factor\;SPF=CF\;\sum \frac{320}{290}\;EE\left(\lambda \right)I\left(\lambda \right)Abs(\lambda )$$

where CF = correction factor (10), referring to an SPF = 4; EE(λ) = erythemogenic effect of radiation; I(λ) = sun intensity; Abs(λ) = spectrophotometric reading of the absorbance of the extracts.

### 3.5. Statistical Analysis

The assays were performed in triplicate, meaning independent experiments were conducted three times, and the results are expressed as the mean ± standard deviation (SD). The data analysis was performed using Statistica 10 software (StatSoft Inc., Tulsa, OK, USA). ANOVA, followed by a post hoc Tukey’s test was applied to evaluate the in vitro experiments. Results were considered statistically significant when the *p*-value was less than 0.05.

## 4. Conclusions

The present study demonstrated that residues of *Humulus lupulus* female cones generated by the brewing industry constitute a promising source of bioactive compounds that can be recovered through green extraction technologies. GC–MS analysis revealed a chemically diverse profile, with 32 compounds identified in the microwave-assisted extract (EH-MA) and 30 in the ultrasound-assisted extract (EH-UA), mainly comprising terpenes, sesquiterpenes, oxygenated sesquiterpenes, and fatty acids. Both extracts exhibited antioxidant activity in cell-based (TBARS and OxHLIA) and chemical (DPPH, ABTS, and FRAP) assays, with EH-MA generally showing superior performance. Antimicrobial assays demonstrated antibacterial activity (MIC 1–10 mg/mL), as well as antifungal activity (MIC 2–2.5 mg/mL). Additionally, both extracts showed selective antiproliferative effects against Caco-2 and MCF-7 tumor cell lines (GI_50_ = 25 μg/mL). The extracts also effectively inhibited nitric oxide production, with EH-MA presenting the strongest anti-inflammatory effect (IC_50_ = 35 μg/mL), followed by EH-UA (IC_50_ = 55 μg/mL). Furthermore, photoprotective potential was observed, with SPF values of 19 for EH-MA and 18 for EH-UA. Overall, these findings highlight the potential of hop cone residues as a sustainable source of multifunctional bioactive extracts, supporting their valorization and upcycling for potential applications in industries products.

## Figures and Tables

**Table 1:** Characterization of compounds present in the extract of hop (*H. lupulus*) cone residue by GC/MS.

Compounds	#	Relative Area (%)	
		EH-UA	EH-MA
β-Pinene	**1**	53.74	61.40
Cyclohexanol 1-methyl-4-(1-methylethenyl) acetate	**2**	0.24	nd
Limonene	**3**	nd	0.26
β-Phellandrene	**4**	0.25	0.31
1,3,6-Octatriene. 3.7-dimethyl-, (Z)-	**5**	0.39	nd
γ-Terpinene	**6**	0.03	nd
Methyl 6-methyl heptanoate	**7**	nd	0.25
Ciclohexene, 1-methyl-4-(1-methyletilidene)	**8**	0.03	nd
Hexanethioic acid, S-methyl ester	**9**	0.02	nd
1,6-Octadien-3-ol,3,7-dimethyl	**10**	0.48	nd
Linalool	**11**	nd	0.87
*Cis*-p-mentha-1(7),8-dien-2-ol	**12**	0.01	nd
3-Decyn-2-ol	**13**	0.01	nd
Methyl nonylate	**14**	nd	0.11
Methyl 6-methyloctanoate	**15**	nd	0.25
2-Decanone	**16**	0.12	0.73
L-α-Terpineol	**17**	0.01	nd
E-10-Dodecen-1-ol Propionate	**18**	0.01	nd
Geraniol	**19**	0.24	0.53
2-Undecanone	**20**	nd	0.09
Methyl 8-methylnonanoate	**21**	nd	0.15
4-Acetonylcycloheptanone	**22**	nd	0.09
1-Methoxy-3-(2-hydroxyethyl) nonane	**23**	nd	0.04
2-Undecanone	**24**	0.04	0.18
2-Undecanol	**25**	nd	0.16
4-Decenoic acid, methyl ester	**26**	nd	1.71
2,6-Octadienal, 2,6-dimethyl-8-(tetrahydro-2H-2-pyranyloxy)	**27**	nd	0.69
Methyl geranate	**28**	nd	0.73
2,6-octadienoic acid, 3,7-dimethyl-, methyl esters	**29**	0.09	nd
Isobutyric acid, tridecyl ester	**30**	0.02	nd
α-Ilangene	**31**	0.06	nd
2,6-Octadien-1-ol, 3,7-dimethyl-, acetate	**32**	0.04	nd
Geranyl acetate	**33**	nd	0.19
α-Copaene	**34**	0.28	nd
Caryophyllene	**35**	5.0	2.70
β-Cubebene	**36**	nd	7.13
*Cis-*β-Farnesene	**37**	23.79	nd
Humulene	**38**	0.43	0.19
Geranyl propionate	**49**	nd	7.46
γ-Muurolene	**40**	1.24	nd
Azulene, 1,2,3,3a,4,5,6,7-octahydro-1,4-dimethyl-7-(1-methylethernyl)-,[1R-(1α.3aβ.4α.7β)]	**41**	0.98	nd
Cadine-1(10).4-diene	**42**	nd	2.92
Seline-3.7(11)-diene	**43**	nd	1.61
Dihomo-γ-linolenic acid	**44**	nd	0.68
Epiglobulol	**45**	nd	0.21
Neryl (S)-2-methylbutanoate	**46**	nd	0.23
17-Octadecen-14-yn-1-ol	**47**	nd	0.16
8,11,14,17-Methyl eicosatetraenoic acid	**48**	nd	1.49
α-Cadinol	**49**	0.03	nd
17-Octadecynoic acid	**50**	0.07	nd
1.5-Dodecadiene	**51**	nd	0.44
2-Pentadecanone	**52**	0.22	nd
-(+)-Ascorbic acid 2.6	**53**	0.16	nd
Palmitic acid	**54**	nd	0.17
Stigmasterol	**55**	0.02	nd

EH-UA: hop residue extract obtained by ultrasound-assisted extraction; EH-MA: hop residue extract obtained by microwave-assisted extraction; nd: not detected.

**Table 2:** Antioxidant capacity of extracts of hop (*H. lupulus*) cone residue.

Extract	Chemical Assays			In Vitro Cell-Based Assays	
	FRAP (µmol TE/mgDW)	DPPH (µmol TE/mgDW)	ABTS (µmol TE/mgDW)	OxHLIA IC_50_ (µg/mL) Δ*t* = 60 min	TBARS IC_50_ (mg/mL)
EH-UA	944.39 ± 6.62 ^cA^	1425.39 ± 6.33 ^aA^	1234.51 ± 13.41 ^bA^	31.30 ± 1.20 ^B^	2.44 ± 0.61 ^A^
EH-MA	928.78 ± 5.45 ^bA^	1139.77 ± 8.85 ^aB^	1097.22 ± 10.31 ^aB^	22.50 ± 0.90 ^A^	3.67 ± 0.84 ^B^
Trolox	nt	nt	nt	21.80 ± 0.20	0.0058 ± 0.0006
Ascorbic acid	nt	2356 ± 1.5	3388 ± 4.0	nt	nt

EH-UA: hop reEH-UA: hop residue extract obtained by ultrasound-assisted extraction; EH-MA: hop residue extract obtained by microwave-assisted extraction; nt: not tested; Capital letter: statistical difference between extracts against antioxidant method; Lowercase letter: statistical difference comparing each extract with all antioxidant methods (separating chemical and in vitro cell-based assays) by Tukey test. nt: not tested.

**Table 3:** Antimicrobial capacity of hop (*H. lupulus*) cone residue extracts.

Antibacterial/Antifungal (mg/mL)		EH-UA	EH-MA	Streptomycin	Methicillin	Ampicillin	Ketoconazole
*Gram-negative*							
*E. Cloacae*	MIC	5	1	0.007	nt	0.15	nt
	MBC	10	5	0.007	nt	0.15	nt
*E. coli*	MIC	10	10	0.01	nt	0.15	nt
	MBC	>10	>10	0.01	nt	0.15	nt
*P. aeruginosa*	MIC	5	2.5	0.06	nt	0.63	nt
	MBC	>10	>10	0.06	nt	0.63	nt
*S. enterica*	MIC	>10	2.5	0.007	nt	0.15	nt
	MBC	>10	>10	0.007	nt	0.15	nt
*Y. enterocolitica*	MIC	10	10	0.007	nt	0.15	nt
	MBC	>10	>10	0.007	nt	0.15	nt
*Gram-positive*							
*B. cereus*	MIC	>10	5	0.007	nt	nt	nt
	MBC	>10	>10	0.007	nt	nt	nt
*L. monocytogenes*	MIC	>10	10	0.007	nt	0.15	nt
	MBC	>10	>10	0.007	nt	0.15	nt
*S. aureus*	MIC	5	2.5	0.007	0.007	0.15	nt
	MBC	10	5	0.007	0.007	0.15	nt
*Antifungal*							
*A. brasiliensis*	MIC	2.5	2.0	nt	nt	nt	0.06
	MBC	>10	>10	nt	nt	nt	0.125
*A. fumigatus*	MIC	>10	>10	nt	nt	nt	0.5
	MBC	>10	>10	nt	nt	nt	1

EH-UA: hop residue extract obtained by ultrasound-assisted extraction; EH-MA: hop residue extract obtained by microwave-assisted extraction; MIC: minimum inhibitory concentration; MBC: minimum bactericidal concentration; nt: not tested; >: greater than.

**Table 4:** Antiproliferative activity of hop (*H. lupulus*) cone residue extracts.

Extract	Antiproliferative Activity (GI_50_; µg/mL)							
	PLP2	Vero	AGS	SI	Caco-2	SI	MCF-7	SI
EH-UA	>400	>400	50.00 ± 2.00 ^bA^	8	100.00 ± 9.99 ^cB^	4	25.00 ± 1.40 ^aA^	16
EH-MA	>400	>400	50.00 ± 2.60 ^bA^	8	25.00 ± 1.15 ^aA^	16	100.00 ± 10.05 ^cB^	4
Ellipticine	1.4 ± 0.1	1.4 ± 0.1	1.23 ± 0.03	1	1.21 ± 0.02	1	1.02 ± 0.02	1

EH-UA: extract of hops residue by UAE; EH-Ma: extract of hops residue by MAE; Vero: green monkey kidney; PLP2: primary pig liver; AGS: gastric adenocarcinoma; Caco-2: colorectal adenocarcinoma; MCF-7: breast adenocarcinoma; GI_50_: extract concentration responsible for inhibiting cell proliferation by 50%; SI: Selectivity Index; Capital letter, statistical difference between extracts against a single cell line; Lowercase letter, statistical difference comparing each extract in relation to all cell lines by Tukey test *p* < 0.05.

**Table 5:** NO-production inhibition capacity of hop (*H. lupulus*) cone residue extracts.

Extract	NO-Production Inhibition (IC_50_; µg/mL)
	RAW 246.7
EH-UA	55 ± 3 ^b^
EH-MA	36 ± 2 ^a^
Dexamethasone	6.3 ± 0.4

IC_50_ extracts concentration responsible for 50% NO-production inhibition; EH-UA: hop residue extract obtained by ultrasound-assisted extraction; EH-MA: hop residue extract obtained by microwave-assisted extraction; Lowercase letter: statistical difference between extracts against RAW 246.7.

**Table 6:** Photoprotective activity (SPF) of hop (*H. lupulus*) cone residue extracts.

Extract	Concentrations/SPF			
	50 (µg/mL)	100 (µg/mL)	500 (µg/mL)	1000 (µg/mL)
EH-UA	17.50 ± 1.13 ^a^	17.70 ± 1.07 ^a^	17.70 ± 1.02 ^a^	17.90 ± 1.02 ^a^
EH-MA	18.40 ± 1.56 ^a^	18.40 ± 1.34 ^a^	18.40 ± 1.27 ^a^	18.80 ± 1.19 ^a^

EH-UA: hop residue extract obtained by ultrasound-assisted extraction; EH-MA: hop residue extract obtained by microwave-assisted extraction; Lowercase letter: statistical difference.

## Data Availability

Data will be made available on request.

## Supplementary Materials

The following supporting information can be downloaded at https://www.mdpi.com/article/10.3390/plants15070994/s1, Figure S1: Chromatogram of the extract from the residue of the hop cone (*H. lupulus*) by GC/MS. (a) EH-UA: hop residue extract obtained by ultrasound-assisted extraction; EH-MA: hop residue extract obtained by microwave-assisted extraction.
