# Supplementary material for: Exploring the Potential of Hop (Humulus lupulus) Cone Residue: Chemical Characterization and Evaluation of Bioactivities
Source: Plants (Basel). 2026 Mar 24;15(7):994. doi: 10.3390/plants15070994 (PMC13074777; doi:10.3390/plants15070994)
Supplement: Supplementary file 1 [file plants-15-00994-s001.zip › plants-4186983-supplementary.pdf]

Supplementary Material

Exploring the potential of hop (Humulus lupulus) cone residue: chemical characterization and evaluation of bioactivities

Giulia Boito Reyes [1], Emylaine Pereira dos Santos [1], Everton da Silva Santos [2], Laura Correia Gonçalves [3], Gabriela Catuzo Canonico Silva [4], Zilda Cristiani Gazim [4], Regina Aparecida Correia Gonçalves [2], Arildo José Braz de Oliveira [2], José Pinela [5,6], Filipa Mandim [5], Tânia C.S.P. Pires [5], Lucio Cardozo-Filho [7], Rúbia Carvalho Gomes Corrêa [1,5] and José Eduardo Gonçalves [1,*]

[1]Programa de Pós-Graduação em Tecnologias Limpas (PPGTL) e Instituto Cesumar de Ciência, Tecnologia e Inovação (ICETI), Universidade Cesumar (Unicesumar), Maringá, Brasil.

[2]Programa de Pós-Graduação em Ciências Farmacêuticas (PCF), Universidade Estadual de Maringá (UEM), Maringá, Brasil.

[3]Departamento de Medicina, Universidade Estadual do Centro-Oeste (Unicentro), Guarapuava, Brasil

[4]Programa de Pós-Graduação em Biotecnologia Aplicada à Agricultura e Programa de Pós-Graduação em Ciência Animal com ênfase em Produtos Bioativos, Universidade Paranaense, Umuarama, Brasil;

[5]CIMO, LA SusTEC, Instituto Politécnico de Bragança, Campus de Santa Apolónia, 5300-253 Bragança, Portugal.

[6]National Institute for Agricultural and Veterinary Research (INIAV, I.P.), Rua dos Lágidos, Lugar da Madalena, 4485-655 Vairão, Vila do Conde, Portugal

[7]Departamento de Engenharia Química, Universidade Estadual de Maringá (UEM), Av. Colombo, 5790, 87020-900 Maringá, PR, Brasil.

*Corresponding author: Universidade Cesumar (Unicesumar) e Instituto Cesumar de Ciência, Tecnologia e Inovação (ICETI), Av. Guedner, 1610, Maringá 87050-39, PR, Brasil; e-mail: jose.goncalves@unicesumar.edu.br.

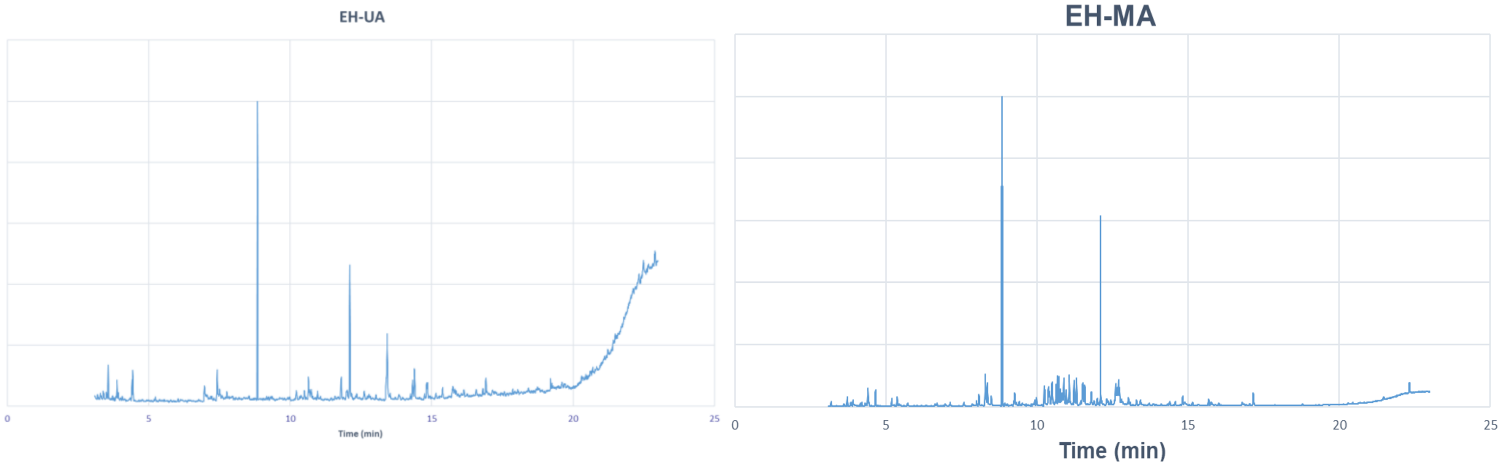

Figure S1: Chromatogram of the extract from the residue of the hop cone (*H. lupulus*) by GC/MS. a) EH-UA: hop residue extract obtained by ultrasound-assisted extraction; EH-MA: hop residue extract obtained by microwave-assisted extraction.
